# 

**Published:** 1816-03

**Authors:** 


### MONTHLY CATALOGUE OF MEDICAL BOOKS.

A SYSTEM of Materia Medica and Pharmacy; including Translations of the London, Edinburgh, and Dublin Pharmacopœias. By John Murray, M.D. Third edition, in 2 vols. 8vo.—Longman and Co.

The Edinburgh New Dispensatory. By Andrew Duncan, jun. M.D. The eighth edition; 8vo.—Longman and Co.

A General System of Toxicology; or a Treatise on Poisons drawn from the Mineral, Vegetable, and Animal Kingdoms; considered as to their Relations with Physiology, Pathology, and Medical Jurisprudence. By M. P. Orfila. Translated from the French. Vol. I. Part 2. 8vo.—Cox and Son.

Dr. Cullen's Practice of Physic, with Notes, explanatory and practical. By Robert John Thornton, M.D. 12mo.—Cox and Son.

A Compendium of Anatomy, Human and Comparative, intended principally for the use of Students. 4 vols. 8vo. with plates. The sixth edition, enlarged and improved. By Andrew Fyfe.

An Epitome of Juridical or Forensic Medicine, for the Use of Medical Men, Coroners, and Barristers. By George Edward Male, M.D. 8vo.—Underwood.

Practical Observations on the Cure of Wounds and Ulcers on the Legs, without Rest; illustrated with Cases. By Thomas Whately. Second edition; 8vo.—Callow.

Observations on Gout and Acute Rheumatism; containing an Account of a safe, easy, and effectual Remedy for those Diseases. By C. Wilson, M.D.—Underwood.

---

### *NOTICES TO CORRESPONDENTS.*

*We are obliged to our Bath correspondent for his hint, which, however, he must be aware, is not new. We fear also that the manner in which he has introduced the preface to a popular work, however meritorious that work may be, will have the air of a puff direct.*

Mr. NORMAN's *promised reply to Dr. Kinglake is received; and we regret that it appears to some of us too severe in a few expressions. Also Communications from* Dr. BERNE, Mr. R. WALKER, AN ARMY SURGEON, A. Z., J. V., *&c. &c. have come to hand.*

